# Unmasking the burden of mental health symptoms and risk behaviors in Vietnamese adolescents: evidence from a multicenter cross-sectional study involving 2,631 high school students

**DOI:** 10.1007/s00127-025-03043-7

**Published:** 2026-01-09

**Authors:** Truc Thanh Thai, Hong-Tuyet Vo Le, Trang Thi Nguyen, Ngon Van Dinh, Xuan Le Mai, Hoai-Thuong Thi Tran, Ngoc-Bich Thi Nguyen, Khanh-Ha Mai Huynh, Thu-An Thi Nguyen, Hy-Han Thi Bui, Minh Cuong Duong

**Affiliations:** 1 Faculty of Public Health, University of Medicine and Pharmacy at Ho Chi Minh City, 217 Hong Bang, District 5, Ho Chi Minh City, Vietnam; 2 School of Population Health, UNSW Sydney, Building C29 HTH Level 5, Sydney, NSW 2052 Australia

**Keywords:** Mental health, Health risk behaviors, Adolescents, High school, Upper secondary school, Vietnam

## Abstract

**Background:**

Adolescents frequently experience symptoms of mental disorders (SOMD) and engage in health risk behaviors (HRB), both of which significantly contribute to global disability and mortality. Despite this, data on these issues remain limited in low- and middle-income countries (LMICs), including Vietnam. This study aims to estimate the prevalence of SOMD and HRB and examine their associations among high school students in Vietnam.

**Methods:**

A cross-sectional survey was conducted with 3,025 students from four high schools and four continuing education centers across Ho Chi Minh City. Participants completed a self-administered questionnaire capturing demographic information, HRB (using the YBRS scale), and SOMD (using the DASS-21 screening scale). SOMD assessments focused on symptoms of depression, anxiety, and stress rather than clinical diagnoses, while HRB covered substance use, risk-taking, physical fighting, suicidal ideation, unsafe sexual behaviors, unhealthy diet, physical inactivity, and sleep deprivation.

**Results:**

Of the 2,631 students included in the analysis, prevalence rates were 42.6% for symptoms of depression, 50.3% for symptoms of anxiety, and 31.1% for symptoms of stress. Engagement in HRB varied widely, from 4.0% for unsafe sexual behaviors to 79.9% for physical inactivity, with 91.6% reporting involvement in multiple HRB. Students experiencing SOMD were significantly more likely to engage in HRB compared to those without SOMD, with odds ratios ranging from 1.24 to 4.64.

**Conclusion:**

SOMD and HRB represent dual and interrelated challenges among Vietnamese adolescents, underscored by their high prevalence. These findings emphasize the critical need for integrated interventions addressing both mental health symptoms and health risk behaviors, especially in resource-constrained LMIC settings.

**Supplementary Information:**

The online version contains supplementary material available at https://doi.org/10.1007/s00127-025-03043-7.

## Introduction

Although adolescence is a crucial stage for the development of behavior, the influence of various pressures such as academic demands, life burdens and social interactions, can increase the risk of involvement in health risk behaviors (HRB) [1, 2]. Health risk behaviors are activities that increase the incidence of disease or injury, potentially leading to disability, mortality, and other societal issues [3]. Behaviors such as tobacco and alcohol use, unsafe sexual behaviors, unhealthy diet, physical inactivity, violence, and risky road behaviors are commonly observed during this stage of development [4, 5]. Data from the US Centers for Disease Control and Prevention (US CDC) Youth Risk Behavior Surveys show a notable rise in the prevalence rates of substance use (0.8% to 30.1%), unsafe sex (5.0% to 31.9%), unhealthy diet (3.1% to 18.0%), inadequate physical activity (30.3% to 52.9%), and suicide (0.7% to 12.5%) [6]. Research conducted in low- to middle-income countries (LMICs) has shown a significant proportion of adolescents demonstrating inadequate physical activity (up to 70.3%), unhealthy eating habits (60.9%), tobacco use (3.1%), alcohol consumption (15.6%), and unsafe sex (6.9%) [7]. Therefore, identifying the full spectrum of HRB in adolescence, particularly within LMICs such as Vietnam, is a critical first step toward tailored prevention and intervention programs.

Although previous studies typically focus on one type of HRB at a time, these behaviors in real life are interconnected and tend to co-exist. This results in more pronounced debilitating outcomes collectively than each behavior could yield independently [8, 9]. Moreover, involvement in any HRB increases the risk of involvement in other HRBs [10]. This phenomenon is demonstrated by Jessor’s theory of behavior-related problems, which suggests that when adolescents engage in multiple risk behaviors, it’s because these behaviors are interconnected and influenced by their environment [11]. For example, results from a cross-sectional study conducted in 11 districts in Malaysia revealed that over 80% of adolescents simultaneously engaged in two or more health risk behaviors [12]. Another study conducted with students aged 11 to 19 in Ghana reported a considerable number of students (94.8%) engaging in multiple HRBs [13]. Consistently high prevalence was also reported in high-income countries like the United States, the United Kingdom, and the Netherlands, with 50% − 70% of individuals participating in two or more HRBs [8]. While interventions may prevent these behaviors, the challenge lies in addressing multiple risk behaviors at once, and that is an urgent call for more comprehensive strategies. Simultaneously targeting multiple HRBs has been shown to be cost-effective, which is vital in resource-limited settings such as Vietnam [14]. 

Apart from HRB, symptoms of mental disorders (SOMD) often emerge during adolescence and if left undetected, they can worsen and have lasting impacts on overall health into adulthood [15]. Approximately 1 in 7 adolescents (around 14%) over the world have SOMD, with depression and anxiety being prevalent disorders causing illness and disability in youth [16]. Stress is also acknowledged as a threat to healthy development during this period [17]. Unfortunately, these SOMD often go unnoticed and untreated, leading some adolescents to engage in HRBs as a coping mechanism for negative emotions [18, 19]. This aligns with stress-coping frameworks such as the Problem Behavior Theory and the self-medication hypothesis, which posit that adolescents may adopt maladaptive behaviors (e.g., substance use, unsafe sex, unhealthy eating) to manage distress or assert autonomy. For example, studies have shown that SOMD are closely tied to thoughts of suicidal ideation [20–22]. Thus, adolescents are not only dealing with SOMD but also at a higher risk of engaging in HRBs. While studies tend to focus on SOMD or HRBs separately, understanding the associations between these problems is essential for improving healthcare services and policies both locally and globally. Unfortunately, data on SOMD and HRB among adolescents in LMICs is limited [1]. 

In Vietnam, several adolescent-support initiatives have been introduced in recent years, including the national School Health Program, the expansion of school-based psychological counseling services, and pilot school-based mental-health promotion interventions [23, 24]. Despite these efforts, implementation remains uneven across regions, and mental-health components are often insufficiently integrated into school settings. As a result, SOMD and HRB in adolescents have not been consistently prioritized in either program delivery or scientific research. Strengthening adolescent mental health in LMICs, including Vietnam, is essential for achieving the Sustainable Development Goals, given the crucial role that this population plays in the future development of these countries [25]. 

Vietnam is a country of diverse cultures, currently opening up to international trade and engaging with various cultures. Understanding the association between SOMD and HRB is essential because emotional distress may directly increase adolescents’ likelihood of engaging in risk behaviors, yet existing studies in Vietnam and other LMICs have rarely examined these problems together. Moreover, rapid changes in academic pressure and digital exposure highlight the need to update prevalence estimates and assess how SOMD and HRB co-occur in the current adolescent population. Also, unique cultural expectations in adolescents such as strong academic competition, intergenerational family obligations, and rapid urbanization create additional pressures that may amplify the vulnerabilities of both SOMD and HRB in this population. Previous studies in Vietnam indicate that both SOMD and HRB among adolescents are at a concerning level, contributing to the leading causes of disability and mortality among Vietnamese adolescents [26–30]. The dynamic environment in Vietnam may influence the difference in the characteristics of SOMD and HRB from other countries and even from historical trends within Vietnam. As the co-occurrence of these problems can compound adverse outcomes and place an even greater burden on adolescents’ physical and mental well-being, examining the links between SOMD and HRB is essential for informing integrated prevention and intervention strategies tailored to Vietnamese adolescents. However, to our best knowledge, no study has been conducted to investigate the associations between SOMD and HRBs. Therefore, this study aims to estimate the prevalence of SOMD, HRBs, and identify the associations between SOMD and HRBs among high school students in Vietnam.

## Methods

### Study setting, design, and participants

Vietnam has 14% of its 97 million population being adolescents [31]. Ho Chi Minh city (HCMC) is one of the two leading economic, cultural and educational cities in the country, consists of 16 districts, five suburban districts and one satellite city. The national education system in Vietnam is divided into various levels with different types of schools. In Vietnam, the high school education includes grades 10 to 12, with grade 10 typically corresponding to 15 years old and grade 12 to 18 years old (with some exceptions for students who repeat a grade or follow alternative educational pathways). In HCMC, there are over 200 high schools and 32 continuing education centers [32, 33]. Students must take a transition examination (i.e. from secondary school to high school) to determine their enrollment eligibility for high schools. In addition, there is another system in place to ensure the goal of universal education in the country which includes one continuing education center in each district. The continuing education centers in HCMC only require completing the basic education program, thus accommodating individuals of varying ages, including those aged more than 18 years and social backgrounds. This form of education provides flexibility in format, study schedules, and curriculum, presenting a tailored approach to meet the learning needs of learners.

**Fig. 1:**
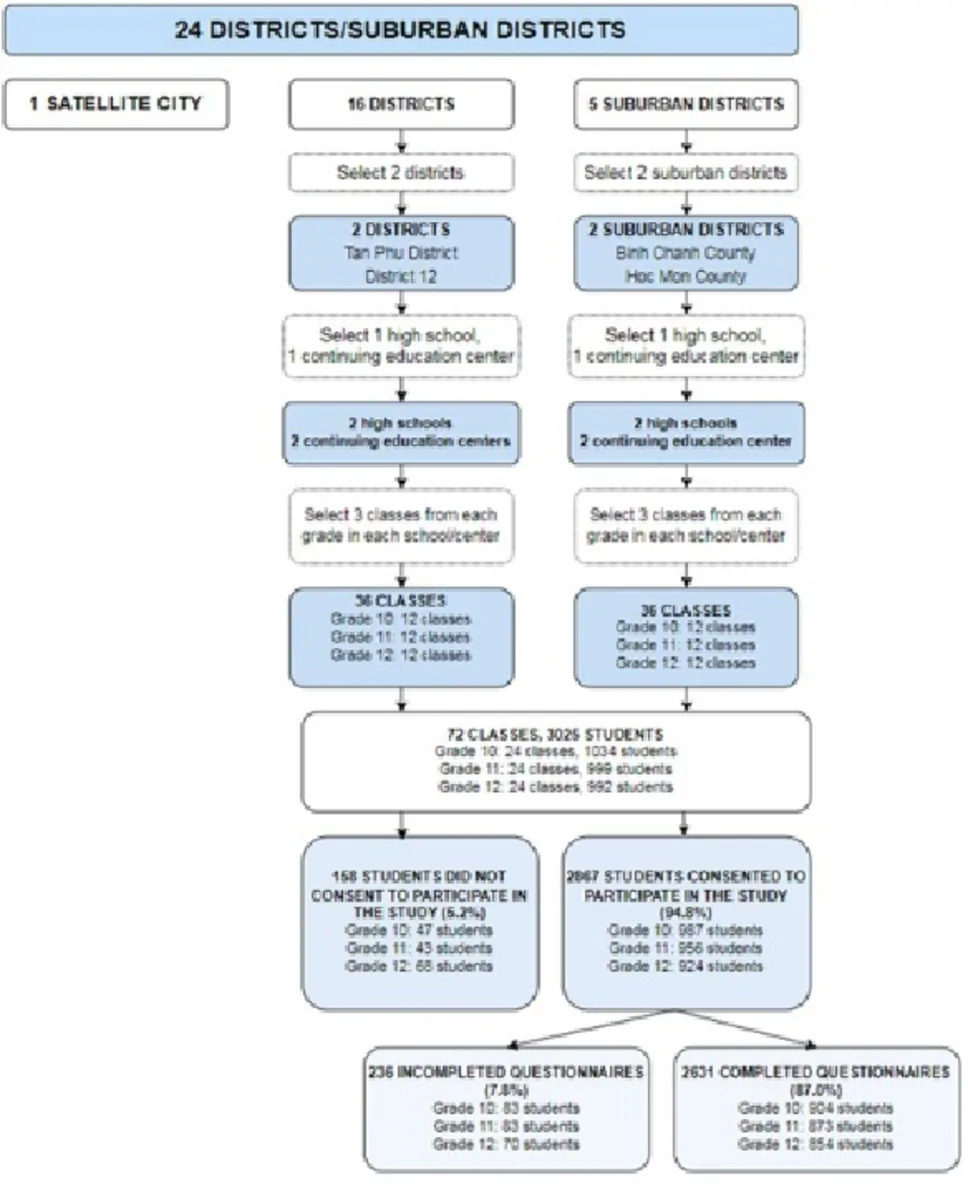
Sampling process

A cross-sectional study was conducted in HCMC, collecting data from February 2024 to April 2024 from students in grades 10, 11, and 12. The multistage cluster sampling method was utilized to recruit participants. Four districts (two urban districts and two suburban districts) were chosen for the study to capture variation in socioeconomic contexts and educational settings, thereby enhancing the representativeness of students across urban and suburban regions of Ho Chi Minh City. Within each district, the continuing education center under the district was selected, along with a randomly chosen high school from that district. Subsequently, three classes from each grade level were chosen from each school/center, resulting in 72 classes participating in the study. The study involved a total of 3,025 students across these classes, with all eligible students invited to participate. Details of the sampling process are presented in Fig. 1.

## Data collection

The research protocol has been approved by the Ethics Committee in Biomedical Research at the University of Medicine and Pharmacy at Ho Chi Minh City (Approval No. 290/HĐĐĐ-ĐHYD and 577/HĐĐĐ-ĐHYD). Participation in the study was entirely voluntary. Parents/guardians provided consent and completed an information sheet first, after which students were asked to provide their written consent to participate in the study. Students who agreed to participate in the study and signed the consent form were provided with a questionnaire and were asked to return the completed questionnaire the next day. As the questionnaires were taken home, this method might introduce measurement biases (e.g., parental influence, peer discussion, or differential response accuracy). Therefore, in this study, several procedures were implemented to minimize these risks, including providing clear written instructions, emphasizing independent completion, and ensuring anonymity.

## Measurement

A self-administered questionnaire included questions about gender (male, female), grade level (grade 10, grade 11, grade 12), having a religion affiliation (yes, no), place of residence (urban, suburban), academic achievement in the last semester (excellent/very good, good, average and below), living with whom (with both parents, with one parent, other), perceived economic status (below average, average, above average), relationship with teachers (poor, normal, good), relationship with friends (poor, normal, good), having academic pressure (yes, no), and frequently experienced health risk behaviors of others (yes, no).

We used the Depression Anxiety Stress Scale (DASS-21) to assess SOMD. This scale consists of 21 items measuring symptoms of depression (7 items, DASS-21-D), anxiety (7 items, DASS-21-A), and stress (7 items, DASS-21-S) in the past week rather than clinical diagnoses. Responses were rated on a Likert scale (0–3 points) based on the frequency of symptoms, with 0 points indicating rarely (< 1 day/week), 1 point for occasionally (1–2 days/week), 2 points for frequently (3–4 days/week), and 3 points for most of the time (5–7 days/week). The total score was then multiplied by 2 to compute individual symptom scores. In this study, the thresholds for assessing symptoms of depression, anxiety, and stress were 14 points, 10 points, and 19 points, respectively. This scale is widely used and has been standardized in Vietnam. The Cronbach’s alpha coefficient of this scale was 0.91 [34].

The study adapted selected items from the Youth Risk Behavior Surveillance (YRBS) scale to evaluate HRB. Developed by the US CDC, this scale evaluates a range of health-related behaviors and experiences among students [35]. Previous studies have shown the strong reliability of this scale [35]. However, the YRBS scale does not define unhealthy dietary patterns based on food consumption and to fill out this gap, we used the Adolescent Food Habits Checklist (AFHC) to assess participants eating behavior. In this study, we assessed eight categories of HRB in the past 12 months, which included (1) substance use, (2) risky transportation, (3) physical fighting, (4) suicidal ideation, (5) unsafe sexual behaviors, (6) unhealthy diet, (7) physical inactivity, and (8) sleep deprivation.

Substance use was defined as students reporting any participation in smoking, drug use, addictive substance use, or alcohol consumption. Risky transportation behaviors were assessed through three specific actions: (1) not wearing helmets/seat belts, (2) being a passenger with a drunk driver and (3) participating in unsafe driving (driving while having been drinking alcohol or using a phone while driving) [36]. Physical fighting was identified when students reported at least one instance of involvement in physical fighting [36]. Students were classified as having suicidal ideation if they reported experiencing it in the past 12 months [36]. Unsafe sexual behaviors were measured by examining five behaviors: (1) having sexual intercourse for the first time before the age of 13, (2) having more than 4 sexual partners throughout their life, (3) drinking alcohol or using drugs before their last sexual intercourse, (4) having sexual intercourse without using a condom, (5) having sexual intercourse without any method to prevent pregnancy [36]. Unhealthy diet was measured using the AFHC scale and based on practice in a previous study, it was defined as having more unhealthy eating behaviors than healthy eating behaviors [29]. Physical inactivity was defined as students being physically active for 4 days or less per week in the past 7 days [37]. Sleep deprivation was classified as sleeping for 7 h or less per night [36]. 

### Data analysis

All analyses were carried out using Stata version 17 (Stata Corp, College Station, TX). Frequency and percentage (%) were used to summarize the distribution of student characteristics, SOMD and HRB. We used Chi-square tests (χ2) or Fishers exact test to evaluate the associations between student characteristics and SOMD or HRB. Logistic regressions were conducted to quantify the associations between SOMD and HRB with and without adjustment for student characteristics. A p-value of less than 0.05 was considered statistically significant.

## Results

### Participants characteristics

A total of 2,631 students were included in the analysis, accounting for 87% of the total number of students invited. Participants’ characteristics are presented in Table 1. There was a comparable distribution between male (46.9%) and female (53.1%). Most students reported not having a religion affiliation (66.6%), living in suburban areas (52.5%) and having an academic achievement of fair or good/excellent level (79.8%), living with both parents (81.0%). More than 80% of students perceived their economic status as normal. In terms of the school environment, most students perceived their relationships with teachers (96.2%) and friends (97.1%) as normal or good. More than half of students (55.7%) reported having academic pressure and 61.6% frequently experienced HRB of others.

**Table 1:** Characteristics of high school students (*N* = 2,631)

Characteristics	Frequency	Percentage (%)
**Gender**		
Male	1234	46.9
Female	1397	53.1
**Grade**		
Grade 10	904	34.4
Grade 11	873	33.2
Grade 12	854	32.5
**Having a religion affiliation**		
Yes	878	33.4
No	1753	66.6
**Place of residence**		
Suburban	1381	52.5
Urban	1250	47.5
**Academic achievement in the last semester**		
Good/Excellent	873	33.2
Fair	1226	46.6
Below Fair	532	20.2
**Living with whom**		
Both parents	2132	81.0
One parent	344	13.1
Others	155	5.9
**Perceived economic status**		
Below average	342	13.0
Normal	2116	80.4
Above average	173	6.6
**Relationship with teachers**		
Not good	99	3.8
Normal	1912	72.7
Good	620	23.6
**Relationship with friends**		
Not good	76	2.9
Normal	1380	52.5
Good	1175	44.7
**Having academic pressure**		
Yes	1465	55.7
No	1166	44.3
**Frequently experience health-risk behaviors of others**		
Yes	1622	61.6
No	1009	38.4

## Symptoms of mental disorders and health risk behaviors

Table 2 presents the frequencies of SOMD and HRB among high school students. Symptoms of anxiety were the most prevalent at 50.3%, followed by symptoms of depression (42.6%) and symptoms of stress (31.1%). Approximately 60% of students had at least one SOMD and nearly one fourth (24.6%) had symptoms of all three SOMD. Physical inactivity and sleep deprivation were the most common HRBs, with prevalences of 79.9% and 78.7%, respectively. More than half of students reported substance use (53.4%) and risky transportation behaviors (56.7%). A total of 542 students (20.6%) reported suicidal ideation in the past 12 months, and 28.8% had an unhealthy diet. Additionally, 105 students (4.0%) reported engaging in unsafe sexual behaviors. Over 90% of participants engaged in multiple behaviors, with 5.5% exhibiting more than five behaviors simultaneously. Details about the association between students’ characteristics and SOMD and HRB are presented in Appendix 1 and Appendix 2. In summary, female students, those reporting academic pressure, poorer relationships with teachers or peers, and those from lower perceived economic status had substantially higher rates of symptoms of depression, anxiety, and stress (Appendix 1). Regarding HRB, male students showed higher prevalence of physical fighting, suicidal ideation, and unhealthy diet, whereas female students had higher rates of physical inactivity and sleep deprivation. Students with lower academic achievement or weaker school connectedness also demonstrated higher involvement in multiple HRBs (Appendix 2).

**Table 2:** Symptoms of mental disorders and health risk behaviors among high school students (*N* = 2,631)

Characteristics	Frequency	Percentage %
**Symptoms of depression**		
Yes	1122	42.6
No	1509	57.4
**Symptoms of anxiety**		
Yes	1324	50.3
No	1307	49.7
**Symptoms of stress**		
Yes	818	31.1
No	1813	68.9
**Number of symptoms of mental disorders**		
0	1080	41.0
1	485	18.4
2	419	15.9
3	647	24.6
**Substance use**		
Yes	1405	53.4
No	1226	46.6
**Risky transportation behaviors**		
Yes	1491	56.7
No	1140	43.3
**Physical fighting**		
Yes	171	6.5
No	2460	93.5
**Suicidal ideations**		
Yes	542	20.6
No	2089	79.4
**Unsafe sexual behavior**		
Yes	105	4.0
No	2526	96.0
**Unhealthy diet**		
Yes	758	28.8
No	1873	71.2
**Physical inactivity**		
Yes	2103	79.9
No	528	20.1
**Sleep deprivation**		
Yes	2071	78.7
No	560	21.3
**Number of health risk behaviors**		
0–1	221	8.4
2–3	1295	49.2
4–5	971	36.9
>5	144	5.5

## Association between symptoms of mental disorders and health risk behaviors

**Fig. 2:**
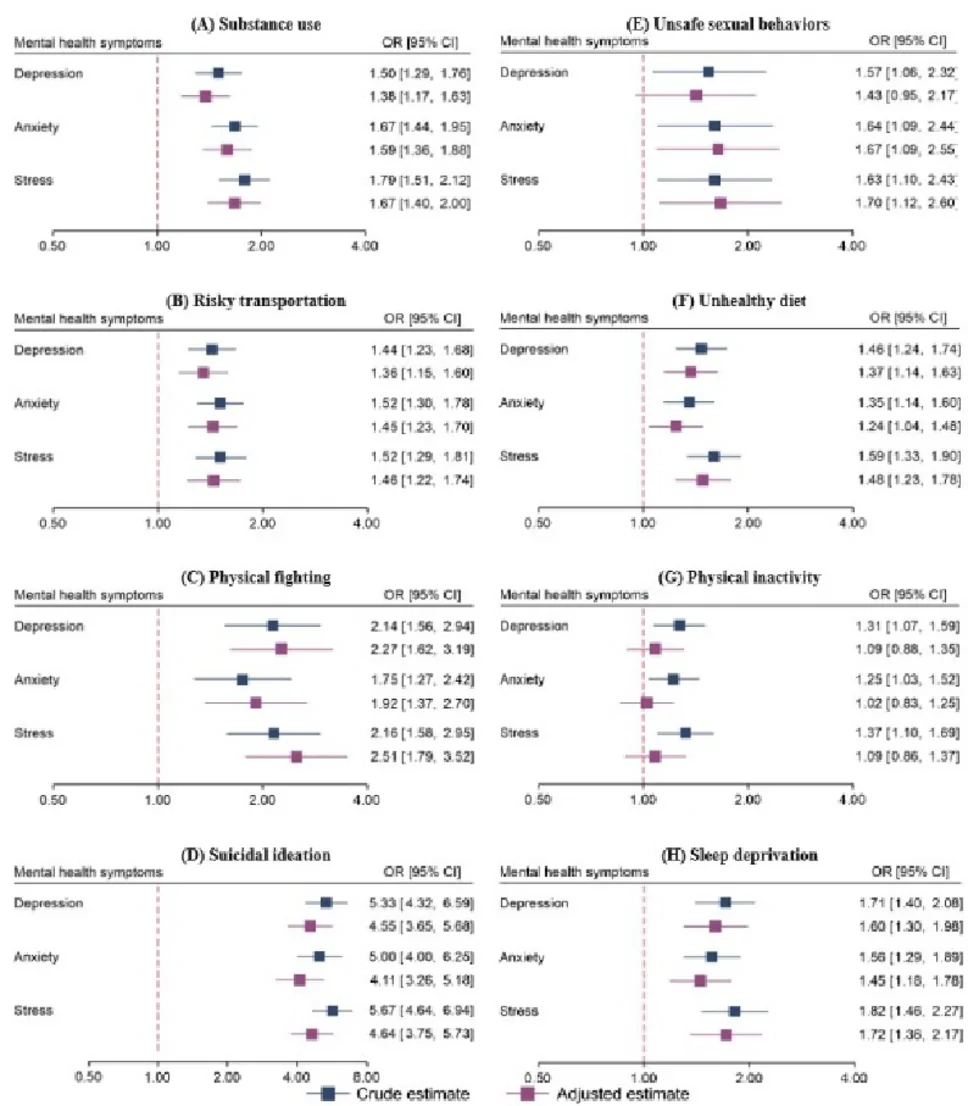
Associations between symptoms of mental disorders and health risk behaviors. Forest plot displaying odds ratios (ORs) and 95% confidence intervals (CIs) derived from univariate (crude estimate) and multivariable (adjusted estimate) logistic regression models. Each square represents the point estimate of the OR for the association between symptoms of depression, anxiety, or stress and each health risk behavior. Horizontal lines indicate the 95% CI for each OR. The vertical reference line at OR = 1 denotes no association. All models were adjusted for gender, grade, having a religion affiliation, place of residence, academic achievement in the last semester, living with whom, perceived economic status, relationship with teachers, relationship with friends, having academic pressure, and frequently experience health-risk behaviors of others

Figure 2 presents the association between each SOMD (i.e., symptoms of depression, anxiety and stress) with each type of HRB with and without adjustment for student’s characteristics. Statistically significant associations were found between all SOMD and all types of HRB, except for physical inactivity. The strongest associations were found between suicidal ideation and symptoms of depression (adjusted OR = 4.55, 95% CI 3.65–5.66), symptoms of anxiety (adjusted OR = 4.11, 95% CI 3.26–5.18), and symptoms of stress (adjusted OR = 4.64, 95% CI 3.75–5.73), while significant associations were also observed between physical fighting and symptoms of depression (adjusted OR = 2.27, 95% CI 1.62–3.19), symptoms of anxiety (adjusted OR = 1.92, 95% CI 1.37–2.70), and symptoms of stress (adjusted OR = 2.51, 95% CI 1.79–3.52). While SOMD was significantly associated with physical inactivity in the univariate analysis, this association was no longer significant after adjusting for student characteristics.

## Discussion

### Key findings

HRB and SOMD have demonstrated their significant burden through numerous studies and are increasingly recognized as major global health concerns. Policy reforms and intervention programs are urgently needed to address these escalating challenges, as their impact can be debilitating and long-lasting, potentially worsening as individuals transition from childhood to adulthood. Our study is among the few that explores the prevalence of SOMD, HRB, and the associations between SOMD and a wide range of HRB in high school students in a LMIC. The results revealed a significant number of students with SOMD and HRB, with a notable association indicating that students with SOMD are more likely to engage in various types of HRB.

The high prevalence of SOMD (i.e., symptoms of depression, anxiety, and stress) found in our study is consistent with a previous study in Vietnam that used the same cut-off points, reporting rates of 34.3% for symptoms of depression, 56.7% for symptoms of anxiety, and 32.8% for symptoms of stress [38]. Studies from other countries used the same scale have also reported SOMD prevalence rates from 33.7% to 78.0% [39, 41]. Although the use of a single scale may not be completely accurate, other studies in Vietnam using different scales, such as the PHQ-9, GAD-7, and PSS-10, have also reported high rates of symptoms of depression, anxiety, and stress among adolescents [42, 43]. These findings underscore the local and global burden of SOMD, as similarly high prevalence rates have been reported in various countries, regardless of the scale used. This suggests that mental health issues among adolescents are a widespread concern, requiring urgent attention and interventions across different cultural and geographical contexts. Moreover, the high prevalence of students in our study exhibited symptoms of all three forms of mental disorders (i.e. symptoms of depression, anxiety, and stress) suggests that mental health challenges in adolescents often manifest as multiple, overlapping conditions rather than isolated issues. Therefore, interventions targeting adolescent mental health need to be comprehensive and multi-faceted, addressing not only one specific disorder but the broader spectrum of mental health.

In terms of HRB, this study revealed a diverse prevalence among Vietnamese adolescents, with physical inactivity and sleep deprivation being the most common. Our findings were consistent with previous studies, highlighted physical inactivity as the most prevalent behavior with rates ranging from 80.4% to 85.5% [12, 44, 45]. Despite the well-documented benefits of physical activity, studies both globally and locally reveal alarmingly high levels of physical inactivity. Furthermore, our results indicated an increasing trend in HRB compared to the Global School-based Student Health Survey (GSHS 2019) in Vietnam, particularly in physical inactivity (75.9%), risky transportation behaviors (15.8%), and suicidal ideation (15.6%) [46]. The increase in suicidal ideation poses a critical concern as adolescents with these thoughts are 12 times more likely to attempt suicide [47]. Moreover, risky transportation behaviors are high in Vietnam due to the widespread use of motorbikes, inconsistent enforcement of traffic laws, and cultural norms that may downplay the dangers of unsafe practices such as not wearing helmets or driving under the influence. In addition, more than half of the students in our study reported substance use, which may be driven by peer pressure, easy access to alcohol and tobacco, and societal attitudes toward substance use. The high prevalence of these HRB underscores the urgent need for targeted interventions at the family, school, and community levels to address these HRBs.

Additionally, our study found that over 90% of participants were involved in two or more HRBs, with 5.5% engaging in more than five behaviors. This prevalence surpassed results from previous studies conducted in Vietnam (82.6%) [30], Malaysia (82.6%) [12], and a multinational study based on data from 89 countries (82.4%) [48], but did not significantly differ from findings in Ghana (94.7%) [13] and data collected from 9 countries in Africa (89.2%) [49]. Differences in reported rates could be attributed to variations in participant demographics, study timing, assessment instruments, the type and the number of behaviors surveyed, cut-off point, and definitions of risk behaviors. The simultaneous engagement in multiple HRBs can raise significant concerns, indicating a potential persistence of harmful behaviors into adulthood and lead to more severe health outcomes [50]. Our study emphasizes the significance of addressing multiple HRB concurrently, rather than focusing on individual behaviors in isolation. This comprehensive approach is especially critical in resource-limited settings like Vietnam, where targeted, multi-faceted interventions can maximize the effectiveness of public health efforts.

Our findings revealed significant associations between SOMD and HRB, except for physical inactivity, which is consistent with findings from previous studies [20–22, 51, 52]. The lack of a significant association between SOMD and physical inactivity may suggest that physical inactivity remains consistently high among adolescents, regardless of their mental health status. This could imply that factors other than mental health, such as lifestyle, environmental influences, or cultural norms, are driving the high levels of physical inactivity, making it a persistent issue even in the absence of mental health symptoms. In addition, descriptive patterns in Appendices 1 and 2 show that physical inactivity was prevalent across nearly all subgroups resulting in limited variability for detecting meaningful associations. Gender appears to be an important confounder, as females reported both higher rates of SOMD and higher levels of physical inactivity, suggesting that the crude association may be partially explained by gender rather than an independent effect of SOMD. School-related factors such as academic pressure and relationships with teachers and peers, which were strongly associated with SOMD but showed weaker or inconsistent patterns with physical inactivity, may have further attenuated the adjusted association. Together, these patterns indicate that physical inactivity in this population may be shaped more by structural or curricular constraints than by SOMD alone.

In contrast, our findings highlight the significant contribution of SOMD to many types of HRB. A large body of literature has explored the mechanisms through which SOMD might affect HRB among adolescents [18, 22]. Adolescents with SOMD may engage in HRB as a coping mechanism, self-medication, or a way to escape emotional distress, diverting their attention from negative emotional challenges. This further strengthens the link between SOMD and HRB, as these behaviors may offer temporary relief but ultimately worsen long-term psychological and physical health. For example, SOMD can intensify feelings of hopelessness, which helps explain their strong association with suicidal ideation. In addition, adolescents involved in physical fighting may be using aggressive behavior as an outlet for underlying emotional distress, or their engagement in violent activities could be exacerbating their mental health challenges. Some studies suggest that SOMD could exacerbate unhealthy diets as individuals seek comfort through the release of neurotransmitters triggered by the consumption of high-sugar and high-fat foods [53]. These findings emphasize the intertwined nature of SOMD and HRB, suggesting the need for integrated intervention strategies that address both mental health and behavioral issues to prevent adverse outcomes.

A high and co-occurring burden of SOMD and HRB among Vietnamese adolescents found in our study suggests multiple practical and theoretical implications. First, schools should implement integrated screening and stepped care such as brief, routine DASS-21 screening within school health services, clear referral pathways to youth-friendly counseling, and protocols for suicidal ideation risk assessment and crisis management, ensuring confidentiality and cultural sensitivity. Second, because many adolescents in our study reported multiple HRB, programs should be multi-component rather than single-issue: sleep hygiene, physical-activity promotion, nutrition literacy, safe transportation practices, substance-use prevention, and non-violent conflict skills. Delivery can combine teacher-facilitated and peer-led sessions, with digital micro-interventions to extend reach, and tailoring for continuing education centers. Third, interventions should engage families and address contextual stressors including academic pressure, intergenerational obligations, rapid urbanization. In Vietnam, academic pressure driven by high-stakes examinations and strong societal expectations, together with intergenerational family obligations that require adolescents to balance schoolwork with household responsibilities, creates culturally specific stressors that may intensify both SOMD and HRB. Previous Vietnamese studies have similarly documented that examination pressure, parental expectations, and family role obligations are among the strongest predictors of stress and depression in high-school students [28, 54]. Therefore, parent psychoeducation on supportive communication, realistic expectations for academic achievement, and healthy digital-media practices can reduce triggers for both SOMD and HRB. Fourth, at the policy level, integrate mental health and HRB modules into the national school health curriculum, train teachers/counselors as gatekeepers, and use task-sharing with community health workers to expand coverage cost-effectively in LMIC settings. Establish standardized monitoring (periodic school surveys and dashboards) to track trends and program performance. Finally, theoretically, the strong SOMD - HRB associations extend Problem Behavior Theory and the self-medication hypothesis to a rapidly modernizing Southeast Asian context. The non-association with physical inactivity after adjustment suggests partially distinct determinants, motivating models that incorporate cultural moderators and environmental constraints.

### Limitations

There are several limitations to consider in this study. First, using a self-report questionnaire on sensitive topics like sexual relationships, substance use, and suicidal ideation may lead to concerns about judgment, social stigma, and underreporting. Second, the need for participants to recall information over varying periods, ranging from 1 week to 30 days or 12 months, raises concerns about the impact of time on recall accuracy. Despite that, previous study [55] has confirmed self-reporting as a prevalent and widely accepted research method. Third, because questionnaires were completed at home rather than in a supervised classroom setting, there is a potential for measurement biases, including parental influence, peer discussion, or inconsistent response accuracy, despite the safeguards implemented to minimize these risks. Fourth, the exclusion of students who don’t have consent from their parents or guardians, as well as those with incomplete questionnaires may have resulted in losing data from Vietnamese adolescents with more SOMD and a higher risk of engaging in HRB. Furthermore, as the study was limited to only one city, its applicability to the entire nation may be limited. In addition, we solely targeted Vietnamese adolescents in schools, potentially overlooking those not in the educational system. To address this, further research across various regions with a more diverse adolescent sample is needed to track differences and trends among different groups. Finally, the cross-sectional design allows us to identify the associations rather than the causality. The ongoing debate persists on whether HRB leads to SOMD, or if it’s the other way around, or if both factors influence each other. This highlights the need for more comprehensive research with intensive follow-up periods to shed light on the associations between these factors.

## Conclusion

Mental Health Problems (SOMD) and Health Risk Behaviors (HRB) are prevalent problems among Vietnamese adolescents, showcasing a concerning co-existence of various SOMD and HRB. Students with SOMD are more prone to involvement in HRB compared to their peers without SOMD. It is essential to develop interventions that target a comprehensive range of SOMD and HRB simultaneously, rather than taking them separately. Regular monitoring through periodic surveys is recommended to track trends and assess the efficacy of prevention programs and interventions.

## Supplementary Information

Below is the link to the electronic supplementary material.

Supplementary Material 1

Supplementary Material 2

## Data Availability

The dataset is not publicly available due to ethical and privacy considerations. However, de-identified data and analysis code may be made available upon reasonable request to the corresponding author, pending approval from relevant institutional review boards and data custodians.
