# Supplementary material for: Unmasking the burden of mental health symptoms and risk behaviors in Vietnamese adolescents: evidence from a multicenter cross-sectional study involving 2,631 high school students
Source: Soc Psychiatry Psychiatr Epidemiol. 2026 Jan 9;61(10):2185–95. doi: 10.1007/s00127-025-03043-7 (PMC13585827; doi:10.1007/s00127-025-03043-7)
Supplement: Supplementary file 1 [file 127_2025_3043_MOESM1_ESM.docx]

**Appendix 1: Association between student’s characteristics and symptoms of mental disorder**

| **Characteristics** | **SYMPTOMS OF MENTAL DISORDER** | | | | |  |
| --- | --- | --- | --- | --- | --- | --- |
|  | **Depression** |  | **Anxiety** |  | **Stress** |  |
|  | **Yes** *n (%)* |  | **Yes** *n (%)* |  | **Yes** *n (%)* |  |
| **Gender** |  |  |  |  |  |  |
| Male | 451 (36.5) |  | 519 (42.1) |  | 285 (23.1) |  |
| Female | 671 (48.0) |  | 805 (57.6) |  | 533 (38.2) |  |
| p-value | <0.001 |  | <0.001 |  | <0.001 |  |
| **Grade** |  |  |  |  |  |  |
| Grade 10 | 380 (42.0) |  | 455 (50.3) |  | 279 (30.9) |  |
| Grade 11 | 366 (41.9) |  | 416 (47.7) |  | 252 (28.9) |  |
| Grade 12 | 376 (44.0) |  | 453 (53.0) |  | 287 (33.6) |  |
| p-value | 0.609 |  | 0.081 |  | 0.102 |  |
| **Having a religion affiliation** |  |  |  |  |  |  |
| Yes | 396 (45.1) |  | 461 (52.5) |  | 285 (32.5) |  |
| No | 726 (41.4) |  | 863 (49.2) |  | 533 (30.4) |  |
| p-value | 0.071 |  | 0.113 |  | 0.283 |  |
| **Place of residence** |  |  |  |  |  |  |
| Suburban | 550 (39.8) |  | 653 (47.3) |  | 411 (29.8) |  |
| Urban | 572 (45.8) |  | 671 (53.7) |  | 407 (32.6) |  |
| p-value | 0.002 |  | 0.001 |  | 0.121 |  |
| **Academic achievement in the last semester** |  |  |  |  |  |  |
| Excellent/Very good | 368 (42.2) |  | 443 (50.7) |  | 274 (31.4) |  |
| Good | 508 (41.4) |  | 608 (49.6) |  | 364 (29.7) |  |
| Average and below | 246 (46.2) |  | 273 (51.3) |  | 180 (33.8) |  |
| p-value | 0.163 |  | 0.766 |  | 0.220 |  |
| **Living with whom** |  |  |  |  |  |  |
| Both Parents | 888 (41.7) |  | 1059 (49.7) |  | 634 (29.7) |  |
| One Parent | 156 (45.3) |  | 175 (50.9) |  | 124 (36.0) |  |
| Other | 78 (50.3) |  | 90 (58.1) |  | 60 (38.7) |  |
| p-value | 0.060 |  | 0.128 |  | 0.007 |  |
| **Perceived economic status** |  |  |  |  |  |  |
| Below average | 185 (54.1) |  | 210 (61.4) |  | 136 (39.8) |  |
| Normal | 864 (40.8) |  | 1031 (48.7) |  | 633 (29.9) |  |
| Above average | 73 (42.2) |  | 83 (48.0) |  | 49 (28.3) |  |
| p-value | <0.001 |  | <0.001 |  | 0.001 |  |
| **Relationship with teachers** |  |  |  |  |  |  |
| Not good | 54 (54.5) |  | 59 (59.6) |  | 36 (36.4) |  |
| Normal | 870 (45.5) |  | 994 (52.0) |  | 619 (32.4) |  |
| Good | 198 (31.9) |  | 271 (43.7) |  | 163 (26.3) |  |
| p-value | <0.001 |  | <0.001 |  | 0.009 |  |
| **Relationship with friends** |  |  |  |  |  |  |
| Not good | 48 (63.2) |  | 46 (60.5) |  | 35 (46.1) |  |
| Normal | 650 (47.1) |  | 732 (53.0) |  | 468 (33.9) |  |
| Good | 424 (36.1) |  | 546 (46.5) |  | 315 (26.8) |  |
| p-value | <0.001 |  | 0.001 |  | <0.001 |  |
| **Having academic pressure** |  |  |  |  |  |  |
| Yes | 750 (51.2) |  | 856 (58.4) |  | 568 (38.8) |  |
| No | 372 (31.9) |  | 468 (40.1) |  | 250 (21.4) |  |
| p-value | <0.001 |  | <0.001 |  | <0.001 |  |
| **Frequently experience health-risk behaviors of others** |  |  |  |  |  |  |
| Yes | 723 (44.6) |  | 873 (53.8) |  | 555 (34.2) |  |
| No | 399 (39.5) |  | 451 (44.7) |  | 263 (26.1) |  |
| p-value | 0.011 |  | <0.001 |  | <0.001 |  |
