# Supplementary material for: Unmasking the burden of mental health symptoms and risk behaviors in Vietnamese adolescents: evidence from a multicenter cross-sectional study involving 2,631 high school students
Source: Soc Psychiatry Psychiatr Epidemiol. 2026 Jan 9;61(10):2185–95. doi: 10.1007/s00127-025-03043-7 (PMC13585827; doi:10.1007/s00127-025-03043-7)
Supplement: Supplementary file 2 [file 127_2025_3043_MOESM2_ESM.docx]

**Appendix 2: Association between student’s characteristics and health risk behaviors**

| **Characteristics** |  | **HEALTH RISK BEHAVIOR** | | | | | | | | | | | | | | | |
| --- | --- | --- | --- | --- | --- | --- | --- | --- | --- | --- | --- | --- | --- | --- | --- | --- | --- |
|  |  | **Substance use** |  | **Risky transportation behavior** |  | **Physical fighting** |  | **Suicidal ideation** |  | **Unsafe sex** |  | **Unhealthy diet** |  | **Physical inactivity** |  | **Sleep deprivation** |  |
|  |  | **Yes** *n (%)* |  | **Yes** *n (%)* |  | **Yes** *n (%)* |  | **Yes** *n (%)* |  | **Yes** *n (%)* |  | **Yes** *n (%)* |  | **Yes** *n (%)* |  | **Yes** *n (%)* |  |
| **Gender** |  |  |  |  |  |  |  |  |  |  |  |  |  |  |  |  |  |
| Male |  | 655 (53.1) |  | 694 (56.2) |  | 107 (8.7) |  | 160 (13.0) |  | 64 (5.2) |  | 309 (25.0) |  | 856 (69.4) |  | 951 (77.1) |  |
| Female |  | 750 (53.7) |  | 797 (57.1) |  | 64 (4.6) |  | 382 (27.3) |  | 41 (2.9) |  | 449 (32.1) |  | 1247 (89.3) |  | 1120 (80.2) |  |
| p-value |  | 0.755 |  | 0.675 |  | <0.001 |  | <0.001 |  | 0.003 |  | <0.001 |  | <0.001 |  | 0.052 |  |
| **Grade** |  |  |  |  |  |  |  |  |  |  |  |  |  |  |  |  |  |
| Grade 10 |  | 444 (49.1) |  | 530 (58.6) |  | 78 (8.6) |  | 200 (22.1) |  | 24 (2.7) |  | 258 (28.5) |  | 725 (80.2) |  | 624 (69.0) |  |
| Grade 11 |  | 496 (56.8) |  | 495 (56.7) |  | 58 (6.6) |  | 170 (19.5) |  | 37 (4.2) |  | 247 (28.3) |  | 686 (78.6) |  | 722 (82.7) |  |
| Grade 12 |  | 465 (54.4) |  | 466 (54.6) |  | 35 (4.1) |  | 172 (20.1) |  | 44 (5.2) |  | 253 (29.6) |  | 692 (81.0) |  | 725 (84.9) |  |
| p-value |  | 0.004 |  | 0.229 |  | 0.001 |  | 0.355 |  | 0.025 |  | 0.810 |  | 0.432 |  | <0.001 |  |
| **Having a religion affiliation** |  |  |  |  |  |  |  |  |  |  |  |  |  |  |  |  |  |
| Yes |  | 473 (53.9) |  | 517 (58.9) |  | 64 (7.3) |  | 184 (21.0) |  | 40 (4.6) |  | 255 (29.0) |  | 696 (79.3) |  | 678 (77.2) |  |
| No |  | 932 (53.2) |  | 974 (55.6) |  | 107 (6.1) |  | 358 (20.4) |  | 65 (3.7) |  | 503 (28.7) |  | 1407 (80.3) |  | 1393 (79.5) |  |
| p-value |  | 0.732 |  | 0.105 |  | 0.245 |  | 0.749 |  | 0.295 |  | 0.852 |  | 0.549 |  | 0.185 |  |
| **Place of residence** |  |  |  |  |  |  |  |  |  |  |  |  |  |  |  |  |  |
| Suburban |  | 743 (53.8) |  | 752 (54.5) |  | 89 (6.4) |  | 281 (20.3) |  | 59 (4.3) |  | 384 (27.8) |  | 1099 (79.6) |  | 1018 (73.7) |  |
| Urban |  | 662 (53.0) |  | 739 (59.1) |  | 82 (6.6) |  | 261 (20.9) |  | 46 (3.7) |  | 374 (29.9) |  | 1004 (80.3) |  | 1053 (84.2) |  |
| p-value |  | 0.666 |  | 0.016 |  | 0.905 |  | 0.736 |  | 0.438 |  | 0.232 |  | 0.636 |  | <0.001 |  |
| **Academic achievement in the last semester** |  |  |  |  |  |  |  |  |  |  |  |  |  |  |  |  |  |
| Excellent/Very good |  | 414 (47.4) |  | 465 (53.3) |  | 37 (4.2) |  | 178 (20.4) |  | 26 (3.0) |  | 244 (27.9) |  | 710 (81.3) |  | 704 (80.6) |  |
| Good |  | 662 (54.0) |  | 688 (56.1) |  | 79 (6.4) |  | 257 (21.0) |  | 42 (3.4) |  | 364 (29.7) |  | 979 (79.9) |  | 963 (78.5) |  |
| Average and below |  | 329 (61.8) |  | 338 (63.5) |  | 55 (10.3) |  | 107 (20.1) |  | 37 (7.0) |  | 150 (28.2) |  | 414 (77.8) |  | 404 (75.9) |  |
| p-value |  | <0.001 |  | 0.001 |  | <0.001 |  | 0.905 |  | <0.001 |  | 0.645 |  | 0.280 |  | 0.111 |  |
| **Living with whom** |  |  |  |  |  |  |  |  |  |  |  |  |  |  |  |  |  |
| Both Parents |  | 1094 (51.3) |  | 1210 (56.8) |  | 137 (6.4) |  | 405 (19.0) |  | 83 (3.9) |  | 605 (28.4) |  | 1706 (80.0) |  | 1671 (78.4) |  |
| One Parent |  | 206 (59.9) |  | 187 (54.4) |  | 19 (5.5) |  | 92 (26.7) |  | 12 (3.5) |  | 99 (28.8) |  | 277 (80.5) |  | 281 (81.7) |  |
| Other |  | 105 (67.7) |  | 94 (60.6) |  | 15 (9.7) |  | 45 (29.0) |  | 10 (6.5) |  | 54 (34.8) |  | 120 (77.4) |  | 119 (76.8) |  |
| p-value |  | <0.001 |  | 0.417 |  | 0.209 |  | <0.001 |  | 0.255 |  | 0.230 |  | 0.706 |  | 0.316 |  |
| **Perceived economic status** |  |  |  |  |  |  |  |  |  |  |  |  |  |  |  |  |  |
| Below average |  | 205 (59.9) |  | 209 (61.1) |  | 35 (10.2) |  | 114 (33.3) |  | 19 (5.6) |  | 101 (29.5) |  | 268 (78.4) |  | 261 (76.3) |  |
| Normal |  | 1096 (51.8) |  | 1183 (55.9) |  | 116 (5.5) |  | 391 (18.5) |  | 74 (3.5) |  | 608 (28.7) |  | 1701 (80.4) |  | 1678 (79.3) |  |
| Above average |  | 104 (60.1) |  | 99 (57.2) |  | 20 (11.6) |  | 37 (21.4) |  | 12 (6.9) |  | 49 (28.3) |  | 134 (77.5) |  | 132 (76.3) |  |
| p-value |  | 0.004 |  | 0.195 |  | <0.001 |  | <0.001 |  | 0.024 |  | 0.945 |  | 0.482 |  | 0.331 |  |
| **Relationship with teachers** |  |  |  |  |  |  |  |  |  |  |  |  |  |  |  |  |  |
| Not good |  | 70 (70.7) |  | 72 (72.7) |  | 14 (14.1) |  | 28 (28.3) |  | 13 (13.1) |  | 32 (32.3) |  | 76 (76.8) |  | 73 (73.7) |  |
| Normal |  | 1024 (53.6) |  | 1090 (57.0) |  | 118 (6.2) |  | 410 (21.4) |  | 68 (3.6) |  | 585 (30.6) |  | 1580 (82.6) |  | 1532 (80.1) |  |
| Good |  | 311 (50.2) |  | 329 (53.1) |  | 39 (6.3) |  | 104 (16.8) |  | 24 (3.9) |  | 141 (22.7) |  | 447 (72.1) |  | 466 (75.2) |  |
| p-value |  | 0.001 |  | 0.001 |  | 0.007 |  | 0.007 |  | <0.001 |  | 0.001 |  | <0.001 |  | 0.015 |  |
| **Relationship with friends** |  |  |  |  |  |  |  |  |  |  |  |  |  |  |  |  |  |
| Not good |  | 48 (63.2) |  | 51 (67.1) |  | 17 (22.4) |  | 28 (36.8) |  | 11 (14.5) |  | 20 (26.3) |  | 60 (78.9) |  | 48 (63.2) |  |
| Normal |  | 756 (54.8) |  | 795 (57.6) |  | 89 (6.4) |  | 300 (21.7) |  | 60 (4.3) |  | 416 (30.1) |  | 1134 (82.2) |  | 1093 (79.2) |  |
| Good |  | 601 (51.1) |  | 645 (54.9) |  | 65 (5.5) |  | 214 (18.2) |  | 34 (2.9) |  | 322 (27.4) |  | 909 (77.4) |  | 930 (79.1) |  |
| p-value |  | 0.042 |  | 0.068 |  | <0.001 |  | <0.001 |  | <0.001 |  | 0.278 |  | 0.010 |  | 0.004 |  |
| **Having academic pressure** |  |  |  |  |  |  |  |  |  |  |  |  |  |  |  |  |  |
| Yes |  | 821 (56.0) |  | 843 (57.5) |  | 87 (5.9) |  | 376 (25.7) |  | 60 (4.1) |  | 436 (29.8) |  | 1185 (80.9) |  | 1226 (83.7) |  |
| No |  | 584 (50.1) |  | 648 (55.6) |  | 84 (7.2) |  | 166 (14.2) |  | 45 (3.9) |  | 322 (27.6) |  | 918 (78.7) |  | 845 (72.5) |  |
| p-value |  | 0.002 |  | 0.312 |  | 0.191 |  | <0.001 |  | 0.758 |  | 0.227 |  | 0.170 |  | <0.001 |  |
| **Frequently experience health-risk behaviors of others** |  |  |  |  |  |  |  |  |  |  |  |  |  |  |  |  |  |
| Yes |  | 906 (55.9) |  | 977 (60.2) |  | 116 (7.2) |  | 379 (23.4) |  | 64 (3.9) |  | 505 (31.1) |  | 1287 (79.3) |  | 1289 (79.5) |  |
| No |  | 499 (49.5) |  | 514 (50.9) |  | 55 (5.5) |  | 163 (16.2) |  | 41 (4.1) |  | 253 (25.1) |  | 816 (80.9) |  | 782 (77.5) |  |
| p-value |  | 0.001 |  | <0.001 |  | 0.085 |  | <0.001 |  | 0.881 |  | 0.001 |  | 0.342 |  | 0.231 |  |
